# Towards Key Principles of Host‐Associated Microbiome Assembly

**DOI:** 10.1111/ele.70433

**Published:** 2026-06-24

**Authors:** Gui Araujo, Torsten Thomas, José M. Montoya, Nicole S. Webster, Miguel Lurgi

**Affiliations:** ^1^ Department of Biosciences Swansea University Swansea UK; ^2^ Centre for Marine Science and Innovation, School of Biological, Earth & Environmental Sciences University of New South Wales Sydney Australia; ^3^ Theoretical and Experimental Ecology Station CNRS Moulis France; ^4^ Institute for Marine and Antarctic Studies University of Tasmania Hobart Australia; ^5^ Australian Centre for Ecogenomics University of Queensland Brisbane Australia

**Keywords:** assembly mechanisms, community ecology, consumer–resource model, dynamical systems, sponge microbiome, symbiosis

## Abstract

Symbiotic relationships between microbes and hosts frequently involve the assembly of complex microbial communities. Community‐level patterns influence life‐history traits, ecological trajectories of partners, and are often critical for host health. These patterns are driven by mechanisms acting at the individual level, including microbial dispersal, host selection, and microbe–resource interactions. Critically, we still lack a clear picture of how these mechanisms interact to shape microbiome assembly. We present a model that describes how distinct community structures arise from those underlying mechanisms. To illustrate the approach, we simulate microbiome data from marine sponges, thereby bridging mechanistic models and empirical patterns. We further apply the model to human data to explore its relevance across systems, proposing that a small set of general mechanisms may govern diverse patterns of diversity and abundance. Our findings advance ecological theory by linking individual‐level processes to community‐scale patterns, illuminating key drivers of microbiome assembly.

## Graphical abstract

Symbiotic relationships between microbes and hosts frequently involve the assembly of complex microbial communities (i.e., the microbiome). Microbiome composition and function influence host life‐history traits, ecological trajectories of partners, and are often critical for host health. Community‐level patterns of microbiome structure are thus driven by ecological processes involving both host and microbes. These processes include microbial dispersal, host selection and microbe–resource interactions. We formulate a theory for how ecological mechanisms generate distinct microbiome community types across hosts. A mathematical model of differential equations grounded on this theory allows us to simulate assembly trajectories of host‐associated microbiomes for different host classifications and compare them. Community‐level patterns of modelled communities recapitulate those observed in sponge and human microbiomes, enabling the identification of key principles behind their assembly.

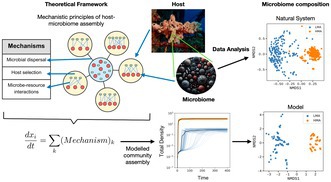

## 1 Introduction

Virtually all multicellular organisms live in close relationships with microbial communities, both influencing and being influenced by them. In many species, including humans, these microbiomes are essential for their health and survival (Dominguez‐Bello et al. 2019). Interactions among hosts, microbes, and their environment occur across spatial and temporal scales, thus shaping the ecological dynamics of host‐microbiome systems (Ferreiro et al. 2018; Nemergut et al. 2013) and the resulting phylogenetic patterns observed in these systems (Fisher et al. 2017; Kolodny et al. 2020; McFall‐Ngai et al. 2013). This interplay between mechanistic processes thus generates systematic differences in microbiome composition among hosts occupying distinct environments. As a result, microbiomes exhibit diverse structures and corresponding functional roles, shaped by their ecological contexts (Lurgi et al. 2019; Pankey et al. 2022; Robinson et al. 2010).

Microbiomes are typically large and diverse microbial communities that assemble throughout a host's ontogenetic development (McFall‐Ngai et al. 2013). Well‐documented examples of joint host‐microbiome development include the human gut microbiome (Guittar et al. 2019), plant rhizospheres (Chaparro et al. 2014) and sponges (Turon et al. 2024). A microbiome's composition is shaped by a variety of biotic (e.g., ecological interactions) and abiotic (e.g., environmental properties) factors, often converging into a characteristic species‐specific structure (Guittar et al. 2019; Martinson et al. 2012; Turon et al. 2024). These factors, or assembly mechanisms, can be quantified and incorporated into theoretical models of microbiome development to investigate their combined effects in shaping patterns of diversity and composition in these microbial assemblages (Araujo et al. 2025; van den Berg et al. 2022). Assembly mechanisms include several processes potentially mediated by the biology and ecology of microbes, host traits, environmental conditions, spatial dynamics and synergies among them (Araujo et al. 2025).

For example, the dispersal of microbes across the host‐environment interface or by horizontal transmission between hosts (Leftwich et al. 2020; Raulo et al. 2024) drives the acquisition of new microbial taxa by the host. Once transmitted, microbial interactions with the host and with other microbes can critically influence their ability to colonise and persist within the microbiome (Mazel et al. 2018; Nappi et al. 2023; Robinson et al. 2010). Colonisation is also governed by selective pressures imposed by the host's internal environment. These pressures can favour the enrichment of specific microbial taxa through host filtering (Mazel et al. 2018) or immune selection (Hooper et al. 2012), especially those beneficial to the host, thus shaping the microbiome's functionality and final composition (Kohl 2020; Mazel et al. 2018; Robinson et al. 2010; Taylor and Vega 2021). Interactions among microbes, and between microbes and hosts, are often mediated by resource exchanges shaped by microbial metabolic traits. These include cross‐feeding among microbes and the bidirectional microbe‐host exchange of resources (Culp and Goodman 2023; Gralka et al. 2020; Pande et al. 2014; Webster and Thomas 2016). Therefore, microbial dispersal, host selection and enrichment, and resource‐based interactions represent key dynamical drivers of microbiome assembly, thus influencing community‐level patterns of microbiome organisation. Quantitative changes in these mechanisms potentially drive distinct community types.

Marine sponges, and their complex microbiomes, exemplify how variation in assembly mechanisms can generate distinct types of microbial communities across host species (Lurgi et al. 2019; Thomas et al. 2016). The ecological and evolutionary processes driving the assembly of marine sponge microbiomes have originated two broad types of sponge species classified by the total cell abundance within their associated microbiomes (Hentschel et al. 2006). Low microbial abundance (LMA) sponge species host relatively sparse microbiomes, whereas high microbial abundance (HMA) sponge species host communities that are over a hundred times denser (Gloeckner et al. 2014; Hentschel et al. 2006). Beyond total microbial load, HMA‐ and LMA‐associated microbiomes differ in other community‐level properties including higher microbial species richness in HMA sponges and marked differences in beta‐diversity between the two groups (Cleary et al. 2025; Lurgi et al. 2019; Moitinho‐Silva, Steinert, et al. 2017). At the host level, HMA sponges exhibit lower pumping rates (Lesser 2023; Rix et al. 2020; Weisz et al. 2008), allocate more resources to their microbial communities (Rix et al. 2020), form tighter functional associations with their symbionts, and more actively enrich specific microbial taxa than LMA sponges (Olinger et al. 2021; Pankey et al. 2022; Weisz et al. 2007). These physiological traits (pumping rate, feeding behaviour and microbial enrichment) enable general mechanisms of microbial dispersal and selection via resource‐mediated processes, thereby shaping microbiome assembly and organisation. Thus, evolved host traits for water pumping and microbial selection could help explain the divergent patterns of abundance and diversity observed between LMA and HMA sponge microbiomes.

Despite extensive knowledge of mechanisms shaping different host‐associated microbiomes, we still lack a unified understanding of how their ecological interplay generates the organisation patterns observed in these complex ecosystems. To this end, theoretical models of community assembly incorporating these mechanisms can help us connect empirical identification of individual processes to system‐level understanding. Although many modelling approaches have recently been developed linking ecological processes to patterns of biodiversity in microbial communities (Camacho‐Mateu et al. 2024; Goldford et al. 2018; Marsland et al. 2020), they focus almost exclusively on interactions between microbes (but see Coyte et al. 2015; Roughgarden 2023; Van Vliet and Doebeli 2019), with little attention given to specific mechanisms of host–microbe interactions. Incorporating host‐centric mechanisms is crucial for understanding the emergence of distinct microbiomes across host or site types, such as HMA versus LMA sponges, or across body sites such as gut and palms in the human microbiome. Identifying a minimal set of assembly mechanisms that operate across biological systems, while imposing system‐specific constraints through host–microbe interactions, would allow a unified and mechanistic characterisation of the emergence of distinct microbiome community structures.

In this paper, we contribute to filling this gap by developing a mechanistic ecological theory for microbiome assembly that incorporates three fundamental processes inferred across host–microbiome systems: microbial dispersal, host selection and microbe–resource interactions. We use this theory to investigate large‐scale patterns of diversity and abundance characteristic of differences in microbiomes of close but distinct community types, thus providing a synthetic view of the classification and emergence of organisation profiles in host‐associated microbial communities. By incorporating empirically observed differences in pumping, enrichment, and microbial feeding across marine sponge types, our model recovers the qualitative differences in microbiome structure, including variation in richness and beta diversity, observed between HMA and LMA sponges. Furthermore, we show that the same model can generalise across systems, as increasing the gap in a single mechanism, while keeping all others unchanged, recapitulates the overall biodiversity shift from sponge (HMA‐LMA) to human (gut‐palm) microbiomes, suggesting a shared underlying ecological structure.

## 2 Model and Results

**FIGURE 1:**
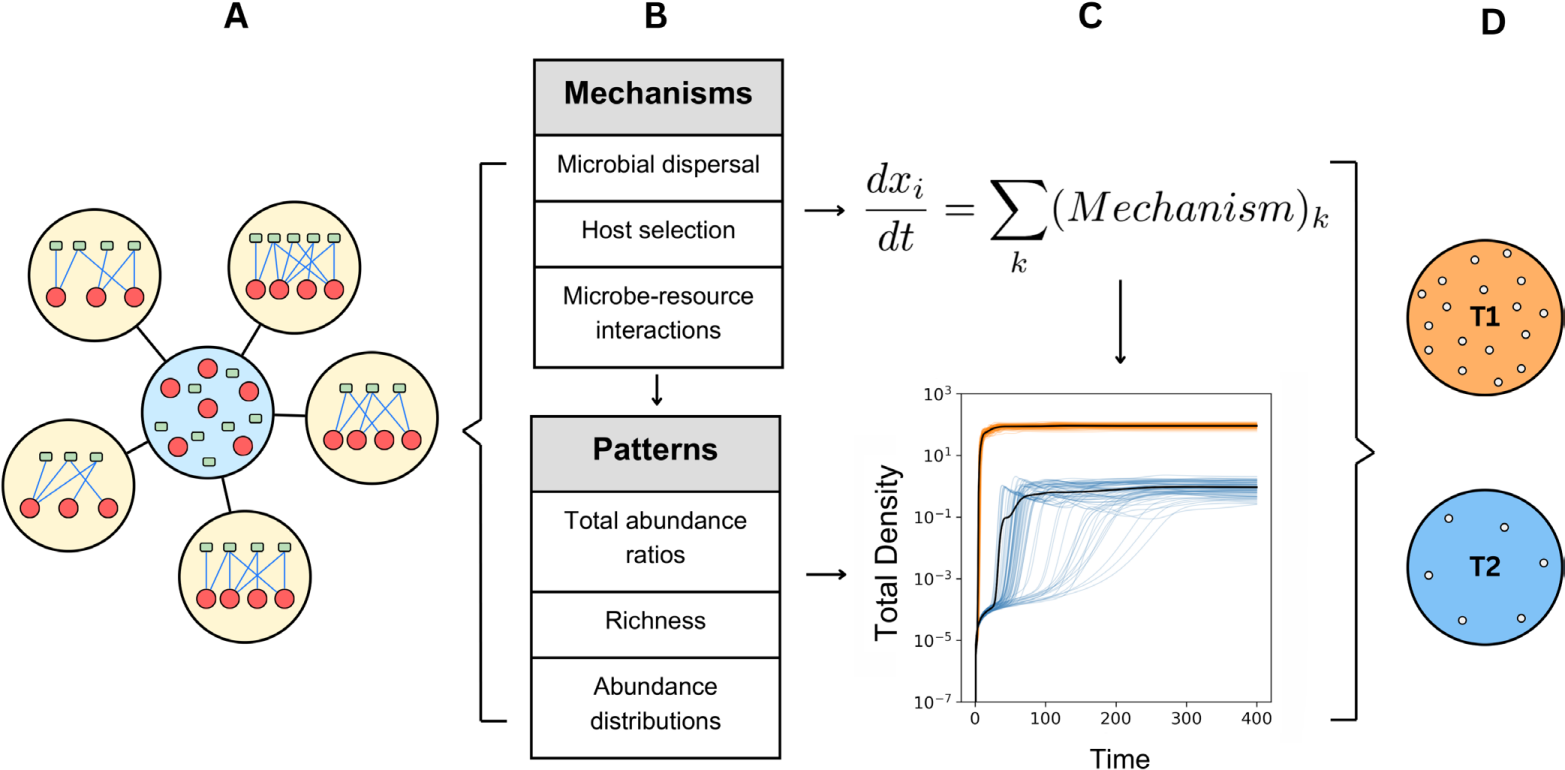
A mechanistic theory for host microbiome assembly. (A) Model setting: Multiple host individuals (large yellow circles) are connected to a shared environment (large blue circle) composed of a constant pool of microbial (red circles) and resource (green squares) types. Each host acquires microbes and resources from the environment and assembles a microbiome represented as a bipartite network linking microbes to the resources they consume and produce. (B) Assembly mechanisms are implemented at the host individual level and drive community‐level patterns of host‐associated microbiomes. Dispersal governs microbial acquisition, host selection shapes differential microbial growth, and resource‐mediated interactions structure microbe–resource network dynamics. (C) The microbiome assembly is modelled through a system of differential equations incorporating various mechanisms and describing temporal changes in microbial and resource densities (microbial counts per volume), or abundances, within each host. Differences in the strength of the assembly mechanisms across hosts generate variation in microbiome community‐level properties, such as species richness and total abundances. Each coloured trajectory represents the assembly of a microbiome within a single host (72 of each type), and the averages are highlighted in black. Different colours represent different relative strengths of the established mechanisms. (D) As a result, hosts can be classified into distinct community types, such as T1 and T2 for high and low total microbial abundance, respectively.

Our microbiome assembly model considers multiple host individuals, each connected to a shared environment composed of a fixed pool of S microbial and R resource types (Figure 1A). Microbial types are distinguished by their dynamical and functional roles within the model, and they more closely refer to functional groups than to Operational Taxonomic Units (OTUs) or species, which differs from the genetic‐based classification in empirical OTU tables. Hosts acquire microbes and resources from the environment and thus assemble their individual microbiomes. Microbes consume and produce resources following a bipartite network of microbe–resource interactions (i.e., different microbe types interact only through resources, see Box 1). The model incorporates three key mechanisms driving microbiome assembly (Figure 1B): (1) microbial dispersal, governing microbial acquisition from the environment; (2) host selection, promoting differential microbial growth within hosts based on their resource production and (3) cross‐feeding interactions between microbial types, in which microbes consume and release resources while relying on the availability of specific resources within the host, resulting in complex interdependencies. The model generates microbiome‐level properties such as total abundance, species richness (i.e., number of distinct microbial types), and species abundance distributions, describing how the interplay among assembly mechanisms gives rise to distinct communities (Figure 1B). To simulate community assembly, we formulate a system of differential equations that tracks changes in the abundance of microbial and resource types within each host (Figure 1C). These equations explicitly represent the three mechanisms alongside terms for microbial death, resource loss, and intraspecific microbial competition (Box 1). As an example of pattern emergence, differences in the relative strength of individual mechanisms, such as increased dispersal or stronger host selection, can lead to substantial variation in total microbial density, or abundance, across hosts (Figure 1C). We then classify hosts and their assembled microbiomes into distinct community types based on emergent features (Figure 1D).

BOX 1 **Ecological model of microbiome assembly.**

Our theory considers individual hosts that import microbial and resource types from a shared environment at a certain rate. Hosts selectively promote the growth of microbes that match their metabolic requirements. Each microbial type consumes a specific set of resources. A fraction of these resources is used for reproduction and the rest is converted into another set of resources interpreted as metabolic byproducts. Let $x_{\mathrm{ih}}$ denote the abundance of microbe i in host h, and $r_{\mathrm{lh}}$ the abundance of resource l in host h. The ecological dynamics within each host are governed by the following system of differential equations:

(1) $$\frac{dx_{\mathrm{ih}}}{dt}=w_{0}S_{\mathrm{ih}}\left(\epsilon \sum_{l^{\prime }=1}^{R}\beta_{il^{\prime }}r_{l^{\prime }h}\right)x_{\mathrm{ih}}-d_{0}x_{\mathrm{ih}}-\gamma x_{\mathrm{ih}}^{2}+\mu_{h}\chi_{i},$$

(2) $$\frac{dr_{\mathrm{lh}}}{dt}=\left(1-\epsilon \right)\sum_{i=1}^{S}\alpha_{\mathrm{il}}\sum_{l^{\prime }=1}^{R}\beta_{il^{\prime }}r_{l^{\prime }h}x_{\mathrm{ih}}-\delta_{0}r_{\mathrm{lh}}-r_{\mathrm{lh}}\sum_{i}\beta_{\mathrm{il}}x_{\mathrm{ih}}+\rho_{l}\eta_{h}\mu_{h}c_{\mathrm{lh}}.$$

where primes denote dummy variables under summation (to avoid confusion with the focal resource l) and each term has a defined ecological interpretation:

1. Microbial reproduction: $w_{0}S_{\mathrm{ih}}\left(\epsilon \sum_{l^{\prime }}\beta_{il^{\prime }}r_{l^{\prime }h}\right)x_{\mathrm{ih}}$: Microbial growth depends on an intrinsic reproduction rate $w_{0}$, the strength of host selection $S_{\mathrm{ih}}$, and the availability of consumed resources. Consumer–resource interactions are determined by consumption rates $\beta_{il^{\prime }}$ of microbe i on resource l, and the effect on growth is summed over all consumed resources. Conversion efficiency of consumed resources into new biomass is given by ϵ.

2. Microbial loss and competition: $-d_{0}x_{\mathrm{ih}}-\gamma x_{\mathrm{ih}}^{2}$: Microbes experience constant mortality at rate $d_{0}$, and intraspecific competition at rate γ, which applies uniformly across microbial types. Interspecific competition is mediated indirectly via shared resources.

3. Microbial dispersal from the environment: $\mu_{h}\chi_{i}$: Microbes from a well‐mixed external pool enter the host at a rate $\mu_{h}$, where $\chi_{i}$ is the constant environmental abundance of microbe i. The model does not assume the continuous introduction of novel microbial taxa over time. Instead, the environmental microbial pool is defined at the outset, and differences in microbiome composition emerge from variation in colonisation success and competitive dynamics among microbes already present in the environment.

4. Production of metabolic by‐products: $\left(1-\epsilon \right)\sum_{i}\alpha_{\mathrm{il}}\sum_{l^{\prime }}\beta_{il^{\prime }}r_{l^{\prime }h}x_{\mathrm{ih}}$: The fraction of consumed resources not used for reproduction is transformed into metabolic products. The rate at which microbe i produces a specific resource l from the total being produced is given by $\alpha_{\mathrm{il}}$, which must satisfy $\sum_{l}\alpha_{\mathrm{il}}=1$. If l is not produced by microbe i, then $\alpha_{\mathrm{il}}=0$.

5. Resource depletion: $-\delta_{0}r_{\mathrm{lh}}-r_{\mathrm{lh}}\sum_{i}\beta_{\mathrm{il}}x_{\mathrm{ih}}$: Resources degrade at a constant rate $\delta_{0}$ and are depleted by microbial consumption, with uptake rates defined by $\beta_{\mathrm{il}}$.

6. Resource uptake from the environment: $\rho_{l}\eta_{h}\mu_{h}c_{\mathrm{lh}}$: Hosts absorb resources from the external environment, where $\rho_{l}$ is the environmental concentration of resource l, $\mu_{h}$ is the host's uptake rate, $c_{\mathrm{lh}}$ the uptake efficiency per resource, and $\eta_{h}$ the fraction of absorbed resources allocated to the microbiome. As a first‐order approximation, we model resource uptake within the same dispersal influx driving microbial uptake (e.g., water pumping in the case of sponges), thus it is proportional to the same rate $\mu_{h}$. This choice explores dispersal intensity as an emergent property of host–environment interactions.

7. Microbe–resource network: To capture resource flow through a simplified metabolic cascade, we imposed a minimal three‐layer structure on the community representing cross‐feeding relations with progressive depletion of nutrient values. We divided microbial types into three functional groups, each corresponding to a different position in the consumer–resource network:

1. The first group consists of microbes consuming primary resources and convert them into metabolic products.
2. The second group consumes these products and produces additional compounds.
3. The third group consumes tertiary resources but does not produce further usable products.

This network organisation defines three corresponding resource sets: primary (externally acquired), secondary (first metabolic products), and tertiary (second‐level products). The consumption (β) and production (α) matrices used in the model are structured into three distinct group blocks.

We assume that all microbial types consume and produce the same number of resources. Although we do not implement specialists and generalists, we aim to capture mean relations in the microbe‐resource network, thus providing a generic structure through which competitive and trophic interactions can emerge.

In our simulations, we assumed three key differences between groups of hosts composed of slightly varying individuals. The first is the microbe and resource uptake rate $\mu_{h}$. The second is the strength of microbial enrichment $S_{\mathrm{ih}}$ through distinct selection capacity $v_{h}$ (Box 2), and the third is the allocation of resources to the microbiome, represented by $\eta_{h}$ as the fraction of acquired resources that is available to microbes within the host. The set of $q_{2}$ non‐essential resources (Box 2) characterises host species. Relatively smaller individual differences within host species were modelled as random variations in trait values (see Section 4).

BOX 2 **Microbial enrichment within hosts.**

Host individuals require resources that are not available in the environment and can only be synthesised by microbes (i.e., secondary and tertiary metabolic products, see Section 4 for details on the microbe–resource network and classification). We assume that all hosts require a shared set of $q_{1}$ randomly defined essential resources drawn from secondary and tertiary resources, which are valued by all host species (universally valued). In addition, each host species is characterised by valuing a particular set of non‐essential resources, drawn randomly from the remaining secondary and tertiary resource pool. Each host has an equal probability of valuing any given non‐essential resource, resulting in an average of $q_{2}$ additional valued resources per host species. This variation in host requirement shapes species‐specific selection pressures on microbial community composition.

Each host has a selection capacity $v_{h}$, defined as the probability of selecting any microbial type that produces at least one resource valued by the host. When a microbial type is selected, its reproductive advantage increases with the number of valued resources it produces. For example, a microbe producing three valued resources is more beneficial, and therefore more strongly enriched, than one producing only a single valued resource. This defines a quantitative, function‐based selection mechanism.

To model the greater adaptive value of microbial specialisation, we assume a superlinear selection curve, where the strength of selection increases faster than linearly with the number of valued resources. If microbe i produces $t_{\mathrm{ih}}$ resources valued by host h, and $\theta_{\mathrm{ih}}$ is a binary variable indicating whether the microbe has been selected ($\theta_{\mathrm{ih}}=1$ with probability $v_{h}$ when i produces at least one resource valued by h, otherwise 0), then the selection factor $S_{\mathrm{ih}}$ affecting microbe *i*'s reproduction in host h is given by:

(3) $$S_{\mathrm{ih}}=1+\theta_{\mathrm{ih}}t_{\mathrm{ih}}^{y}$$

When a microbe is not preferred by the host ($\theta_{\mathrm{ih}}=0$) or does not produce any valued resources ($t_{\mathrm{ih}}^{y}=0$), then $S_{\mathrm{ih}}=1$, meaning no enrichment. y is a parameter that controls the rate of increase in the response of $S_{\mathrm{ih}}$ to the production of valued resources and is set to y=2 in our simulations to implement moderate superlinearity.

### 2.1 Microbial Dispersal, Enrichment and Resource Dynamics Drive the Assembly of Distinct Host Types

We parameterised our model to investigate mechanisms identified by previous studies as potential drivers of sponge microbiome structure, and these mechanisms' ability to recover observed organisational features of these communities. Marine sponges are able to control the import of microbes and resources from the external environment via pumping of seawater, thereby driving microbial dispersal (Turon et al. 2018). Empirical data have shown that LMA sponges exhibit ~2.5 times higher pumping rates ($\mu_{h}$ in our model) than HMA sponges (Rix et al. 2020). We thus incorporated this heterogeneity across host types into our model. In terms of the host selection mechanisms, it is known that HMA sponges can maintain more intimate and functionally specific associations with their microbiomes (Olinger et al. 2021; Pankey et al. 2022; Weisz et al. 2007). We modelled this by allowing differences in the selection capacity of microbes by hosts ($v_{h}$), resulting in a greater probability of enriching microbes that produce valuable metabolic resources in hosts with higher $v_{h}$ values. Finally, substantial differences in the magnitude of resource allocation have been observed across sponge types. HMA sponges typically allocate a greater share of environmental resources to their microbes, consuming less resources directly. To reflect this, in our model, we established 100‐fold differences in the fraction of resources made available to microbes ($\eta_{h}$) between groups of hosts, in line with empirical estimates for differences between HMA and LMA sponges (Rix et al. 2020).

We simulated communities comprising multiple host species of LMA and HMA types (see Section 4 for full specification of parameters), with 72 individuals in each group, divided into 12 species of 6 individuals each, and implementing the distinctions described above across host types for all three mechanisms. We contrasted our model results with empirical patterns of sponge‐associated microbial communities where sponge samples from many species had previously been classified into HMA and LMA categories based on total microbial biomass in host tissues (Moitinho‐Silva, Nielsen, et al. 2017). To make our simulated data comparable to empirical data, we sampled microbial counts proportional to final abundances (24,000 counts per sample), generating simulated host sample versus microbe species tables (equivalent to the Operational Taxonomic Units [OTU] tables commonly analysed in microbiome studies) (Thomas et al. 2016).

**FIGURE 2:**
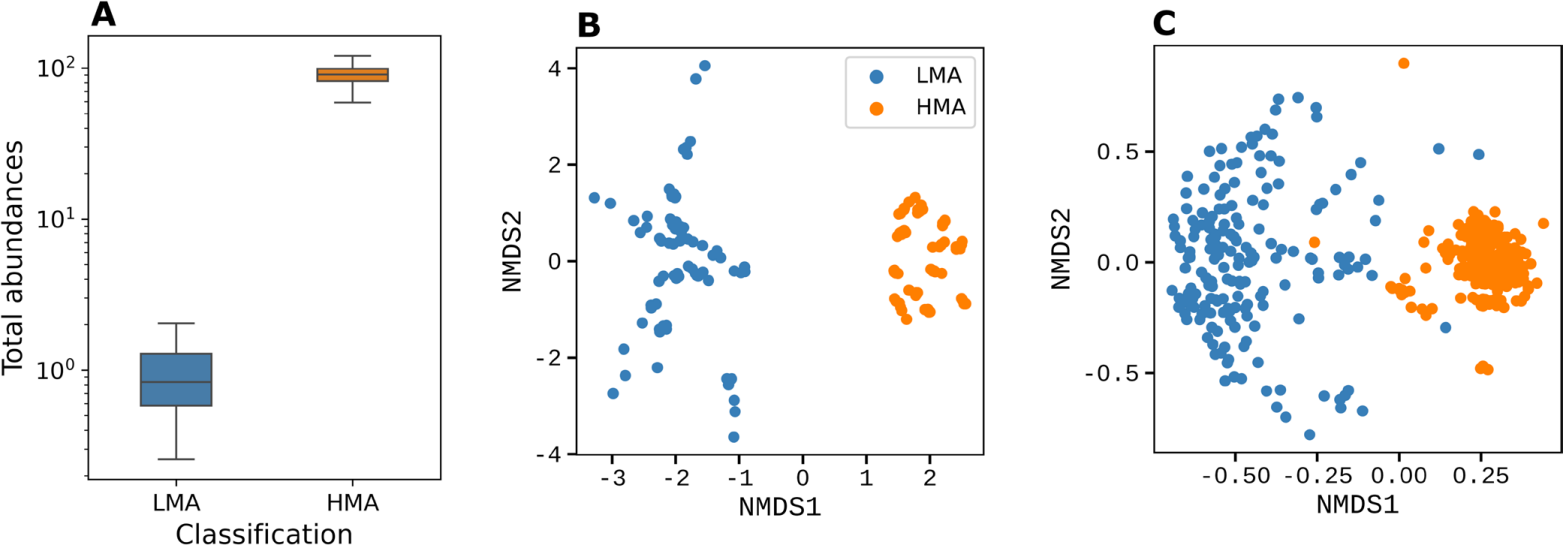
Compositional differences observed between microbiomes of HMA and LMA sponge hosts are determined by microbial dispersal, host selection and enrichment. Comparison of model outputs and empirical data (Operational Taxonomic Unit or OTU tables) of marine sponge samples from the Sponge Microbiome Project (see Section 4 for details): Total abundances (only simulation) and community composition of microbiomes highlighting the separation between groups of host types. (A) Simulations of several LMA‐ and HMA‐type hosts after microbiome assembly reproduce the defining characteristic of the HMA‐LMA separation. Samples' distributions from the model show a difference of two orders of magnitude in the total abundance (or density) of microbes within simulated host individuals separated by their classification (72 samples of each). LMA median = 0.8, HMA median = 90.5. Box plots feature the median line, the boxes denote the interquartile range (IQR), and whiskers denote the rest of the data distribution. (B) Non‐metric multidimensional scaling (NMDS) of Bray‐Curtis dissimilarities in relative abundances of OTU‐equivalent units in the simulated communities (stress = 0.185). OTU‐like tables were generated by sampling modelled microbial types with probabilities proportional to their final abundances. The analysis reveals two well‐separated clusters corresponding to HMA‐ and LMA‐type hosts. (C) NMDS applied to empirical data also shows a clear separation between HMA and LMA samples (stress = 0.178). Rarefaction of the empirical data were set at 23,376 counts (minimum number of reads in the dataset) on 312 HMA and 184 LMA samples divided into 19 HMA and 17 LMA species. The model was sampled at 24,000 counts, generating simulated tables for 72 HMA and 72 LMA samples, equally divided into 12 HMA and 12 LMA species. In the model, LMA‐type hosts had pumping rates 2.5 times higher than HMA‐type hosts, resource allocation to microbes was 100‐fold lower in LMAs, and selection capacity was set to 0.01 for LMA and 0.2 for HMA hosts.

Our model produced a difference of two orders of magnitude in total microbial abundances between HMA and LMA samples, consistent with empirical findings reporting microbiomes at least 100 times more abundant in HMA sponges (Gloeckner et al. 2014; Hentschel et al. 2006) (Figure 2A). Composition of simulated communities also formed two distinct clusters corresponding to HMA‐ and LMA‐type hosts, as revealed by non‐metric multidimensional scaling (NMDS, stress = 0.185) on Bray–Curtis dissimilarities (Figure 2B) and PERMANOVA tests confirming that variation between groups was greater than variation within groups (pseudo‐*F* = 18.5, 999 permutations *p* = 0.001, *R* ^2^ = 11.5%).

This clear separation mirrors that observed in empirical sponge microbiome data, calculated from 312 HMA and 184 LMA samples divided into 19 HMA and 17 LMA species and rarefied to 23,376 reads (Figure 2C) (NMDS stress = 0.178, PERMANOVA tests: pseudo‐*F* = 77.8, 999 permutations *p* = 0.001, *R* ^2^ = 13.6%). A further similarity lies in the within‐group spread: LMA samples showed greater dispersion, whereas HMA samples were tightly clustered in both modelled and empirical data (LMA to HMA ratio of mean distance to centroid of around 1.80 in the entire data, 1.38 in a subsample of data shown in Figure S1, and 1.16 in the model). An analysis of a subsample of the data with a sampling size closer to the model and more homogeneous between groups (7 HMA and 7 LMA species with 6 samples each) reveals no meaningful changes (Figure S1). These results demonstrate that the assembly model successfully reproduces the defining features of the observed dichotomy between HMA and LMA sponge microbiomes, lending support to the hypothesis that the identified mechanisms are likely drivers of assembly in host‐associated microbiomes.

Mechanisms above were tested in isolation to determine their relative contributions to observed patterns. Interestingly, none of the mechanisms alone could reproduce empirical organisation patterns. For instance, we tested differences in pumping rates as the only distinction between host groups, with parameters concerning the other mechanisms remaining equal. This resulted in lower abundance and richness for the HMA‐type hosts (i.e., those with lower pumping rate), contrasting sharply with empirical observations (Figure S2A). Higher resource allocation and selection capacity would then be required to counterbalance the lower pumping rate, allowing the HMA's more stable internal environments and lesser variation across microbiomes (Lurgi et al. 2019; Pankey et al. 2022). We tested these two mechanisms separately counteracting lower pumping rates, which resulted in partial matches to empirical observations (Figure S2B,C). Therefore, the action of both mechanisms together, on top of a lower pumping rate, were necessary to reproduce the patterns seen in natural microbiomes. These tests suggest that hosts with lower pumping rates can absorb less microbes and resources from the environment if they are better able to exert a tighter control on their internal environment, in a trade‐off allowing them to draw more benefits from their microbiome. More generally, this suggests that the patterns distinguishing community types (e.g., composition and abundance) are determined by differences in the contributions of fundamental ecological mechanisms (e.g., dispersal and resource availability), which are in turn driven by system‐specific biological distinctions (e.g., water pumping and host feeding mechanisms).

### 2.2 Beyond Compositional Differences: Species Richness and Abundance Distributions

**FIGURE 3:**
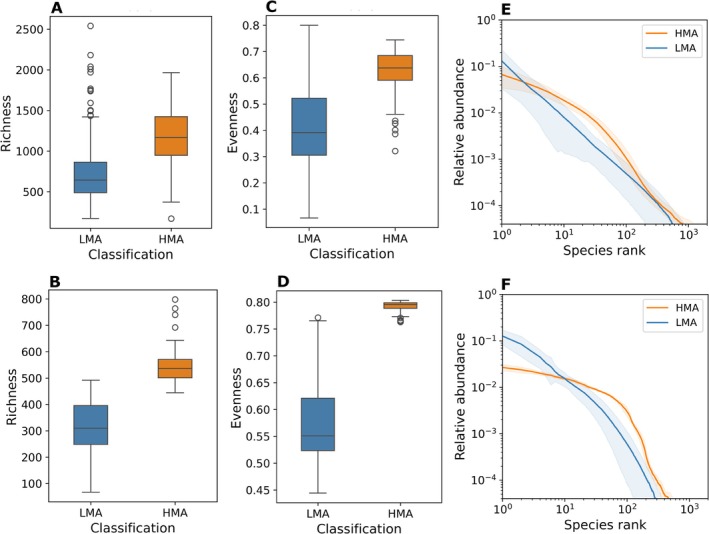
Microbiome complexity is driven by differences in microbial dispersal, selection and enrichment across host types. Comparison of microbiome complexity between empirical data (OTU tables) of marine sponge samples from the Sponge Microbiome Project (top row) and model simulations (bottom row). Box plots show the distribution of host samples generated from the OTU tables, empirical (312 HMA and 184 LMA samples) and simulated (72 HMA and 72 LMA samples), separated by their classification. (A, B) HMA sponges exhibit higher number of OTUs or microbial species richness than LMA sponges in natural samples. The same relationship is preserved in simulations. Although the total pool of OTUs in the model is *S* = 3000, the natural data includes 56,161 OTUs. (C, D) Evenness is lower and more variable among LMA sponges in the empirical data. Model outputs reproduce this pattern, with HMA samples exhibiting consistently higher evenness. (E, F) Rank‐abundance plots of average relative abundances (i.e., the sample averages of each rank) show differences in abundance distributions across host types. HMA hosts distributions present a smaller standard deviation (shaded region, the sample standard deviation of the ranked abundances) and exhibit a more prominent curve of abundances, representing more similar distribution of taxa with higher abundances across both empirical and model data. Simulations were the same as in Figure 2. Box plots feature the median line, the boxes denote the interquartile range (IQR), whiskers denote the rest of the data distribution and outliers are denoted by points greater than ±1.5 × IQR.

Previous analyses of marine sponge microbiomes have revealed that HMA sponges typically harbour more complex microbiomes than LMA sponges, in terms of the absolute number of OTUs per host and the evenness of their abundances (Moitinho‐Silva, Steinert, et al. 2017), a distinction well captured by our analysis of the data (Figure 3A,C) and model (Figure 3B,D). A Welch *t*‐test confirmed the separations in richness (t=15.78,p<0.001 for the model and t=12.28,p<0.001 for the data) and evenness (t=20.63,p<0.001 for the model and t=18.66,p<0.001 for the data). Further, rank‐abundance curves from empirical data reveal greater variation in abundance distributions among LMA sponges (i.e., size of shaded area around the lines in Figure 3E; Moitinho‐Silva, Steinert, et al. 2017). Moreover, HMA microbiomes tend to show slightly greater similarity in the abundances of dominant taxa, with a smaller slope of the curve for higher abundances suggesting a higher degree of shared dominance of abundant microbial types. These trends are also recovered by the model, with the HMA curve being more horizontal than the LMA curve at the beginning (Figure 3F). These results indicate that differences in microbial dispersal, host selection, and resource allocation implemented in the assembly model can jointly reproduce observed patterns of richness, evenness, and abundance distributions in HMA versus LMA microbiomes.

To assess the model's sensitivity to the functional form of the host selection function $S_{\mathrm{ih}}$, we ran simulations for several values of the exponent $y=\left\{0.5,1,1.5,2.5,3\right\}$ and obtained similar results, although the group spread in ordination analysis suggests that values around 2 best reflect the data (Figure S3). We also assessed the sensitivity to the number of fixed and random resources valued by host individuals (Box 2). The main model uses ($q_{1}=15,q_{2}=15$) and we tested different proportions and values: ($q_{1}=15,q_{2}=0$), ($q_{1}=15,q_{2}=30$), ($q_{1}=5,q_{2}=15$), ($q_{1}=30,q_{2}=15$) and ($q_{1}=30,q_{2}=30$). Similar results were obtained in all cases, although the group spread in ordination analysis suggests that similar proportions better reflect the data (Figure S4). We also explored the sensitivity of the total number of microbial types, S (Figure S5). The main model uses S=3000 and we tested different values $S=\left(1000;1500;2000;2500\right)$. Again, similar results were obtained in all cases. Together, these sensitivity analyses (Figures S3–S5) strengthen the reliability of the hypotheses drawn from our model, showing that the observed differences across community types are not conditioned on a specific region of the parameter space.

### 2.3 Unifying Assembly Mechanisms Across Microbiomes

In marine sponges, pumping and microbiome feeding constitute physiological processes through which ecological mechanisms of dispersal and resource dynamics are manifested. Other host‐microbiome systems might differ in biological details but are likely to incorporate processes with similar roles in the generation of mechanisms of acquisition, selection, and microbe–resource interactions. Therefore, the dynamical model developed above should be generalisable to implement mechanisms and capture patterns from microbiome differences observed across host‐microbiome systems. To explore and illustrate this generalisation, we analysed time‐series data extracted from human microbiomes from faeces and palm body sites (Caporaso et al. 2011; Mitchell et al. 2018). We highlighted the shared mechanistic structure underlying sponge and human microbiomes and hypothesised that differences in their empirical biodiversity patterns reflect shifts in the intensity of a common ecological process. Based on the assembly mechanisms proposed in our theory, we expected that palm microbiomes could be fundamentally distinguished from faeces ones due to the former being external to the body, and thus more strongly subjected to environmental influences, as well as not being part of an internal body system (i.e., the digestive system). We hypothesised that, as for the LMA group in sponges, the palm microbiome experiences a higher turnover of microbial cells and smaller resource allocation and selection by the host.

**FIGURE 4:**
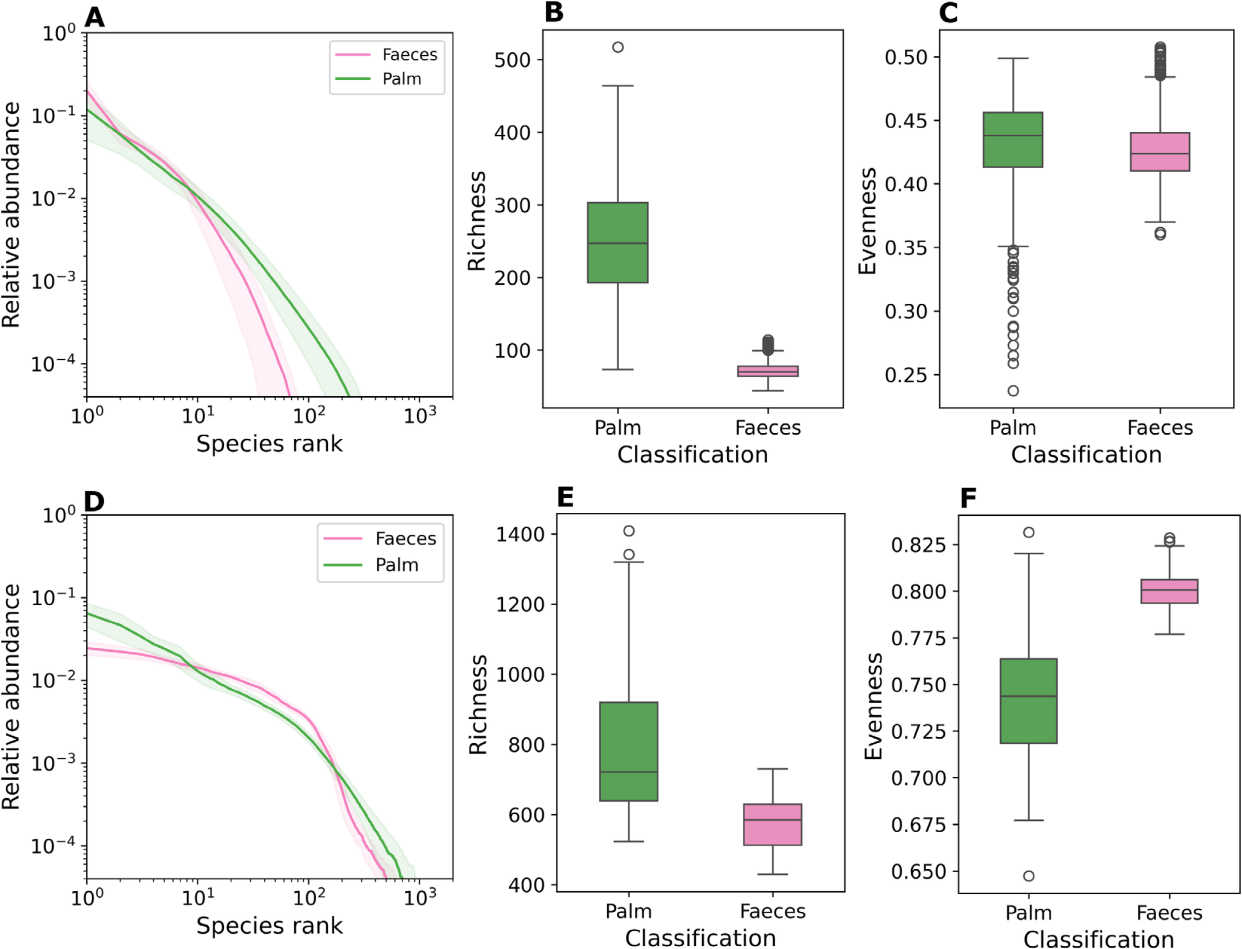
Human microbiome analysis suggests generality of assembly mechanisms across microbiome types. Comparison of different human microbiome sites (top row) with model outputs (bottom row) shows how the same assembly mechanisms, with quantitative parameter changes, can generate distinct microbiome patterns observed across systems. Empirical timeseries data of microbiomes from palm (two sets of 265 and 20 samples) and faeces (330 samples) of a human subject are available from MGnify EBI Metagenomics (see Section 4 for details). Simulations included 72 samples each for palm‐ and faeces‐type communities, ran over 250 time units (sufficient for microbiome assembly in this case). Box plots show the distribution of host samples generated from the OTU tables, empirical and simulated, separated by their classification. (A) Rank‐abundance curves for faeces and two palm (left and right) datasets show their mean relative abundance (solid line, see previous figure for details) and standard deviation (shaded region). Curves are coloured by group. The palm microbiome (external body surface) forms a distinct cluster from the faecal microbiome (internal body environment), which shows similar abundance profiles. (B, C) Palm microbiomes exhibit consistently higher richness and slightly higher evenness than those from the faeces. (D–F) Simulation results approximate the empirical patterns, closely resembling observed trends in relative values and variability. To theoretically investigate empirical patterns of human microbiomes, faeces microbiomes were modelled as having a higher microbial enrichment and resource availability compared to palm microbiomes, analogous to HMA (vs. LMA) sponge hosts. Palm microbiomes were given a 10‐fold higher microbial flux relative to faeces (vs. the 2.5‐fold ratio used for LMA vs. HMA sponges). The data were rarefied with 20,000 reads, and we sampled the model to generate tables with the same number of counts. Box plots feature the median line, the boxes denote the interquartile range (IQR), whiskers denote the rest of the data distribution, and outliers are denoted by points greater than ±1.5 × IQR. See Section 4 for further details.

We analysed the differences in microbial species richness, evenness and abundance distributions between the palm and the faecal microbiomes (Figure 4A–C). However, contrary to the microbiomes of LMA sponges, those of palms comprise the microbiome type with higher richness and evenness when compared to the faeces type (see Figure S6 for explicit comparisons to left and right palms). We used the same model setup as before and only increased the differences in microbial dispersal to palm having 10 times the faeces dispersal instead of 2.5 used for the LMA against HMA sponges. Therefore, we hypothesised a larger difference in microbial dispersal in this case and chose a 10‐fold separation as an exploratory modelling choice. We tested other values of dispersal and obtained similar results (see Figure S7), although values around 10 yield a better representation of the richness separation observed between faeces and palm microbiomes. As a result, this implementation more closely approximates the observed differences across human microbiomes (Figure 4D–F). The model captures the inversion in which richness is larger for the palm microbiome (t=−8.22,p<0.001 for the model and t=−44.92,p<0.001 for the data). Although simulation results for evenness do not match observations (t=12.42,p<0.001 for the model and t=0.48,p=0.63 for the data), the model captures the qualitative difference in variances between palm and faeces (significant differences in variance in Levene's test, p<0.001, for both simulations and empirical data). These results suggest that differences in assembly mechanisms of palm against faeces in human microbiomes and LMA against HMA in sponge microbiomes are mainly represented by a larger difference in microbial dispersal and colonisation from the external pool. This observation could be explained by the human palm being typically exposed to a more diverse array of contributing input environments (i.e., humans touch many different surfaces on a daily basis) as compared to the relatively more constant input of seawater experienced by sponges, given their sessile life. Although the correspondence of absolute richness and evenness values between data and simulations is beyond the scope of our analysis, our results suggest that other drivers of assembly might play equally important roles in both palm and faeces microbiomes which are not present or not as important in sponge microbiomes.

## 3 Discussion

Mechanistic modelling of host‐microbe associations, grounded in quantitative representations of empirically observed mechanisms, offers a powerful theory to describe the emergence of these complex relationships. This is a growing field which in the last decade has revealed fundamental insights into how nature can shape complex microbial associations, both free living (Goldford et al. 2018; Hu et al. 2022; Lopes et al. 2024) and host‐associated (Camacho‐Mateu et al. 2024; Grilli 2020; Van Vliet and Doebeli 2019). This work has revealed underlying general mechanisms of microbial interactions that can result in connectivity patterns, and their dynamical consequences, enhancing the persistence of microbial assemblages. In host‐microbiome associations, however, specific controlling mechanisms of the host are hypothesised to influence microbiome dynamics (Araujo et al. 2025; Coyte et al. 2015; Pankey et al. 2022; Rix et al. 2020). Incorporating these processes into a general theory of host‐associated symbioses is fundamental to better understand their formation.

We introduced a general ecological model and theory of community assembly linking host‐microbiome mechanisms to emergent patterns of microbiome organisation and diversity. The model incorporates microbial dispersal, host selection and enrichment, and resource‐mediated microbial interactions as fundamental assembly mechanisms. These factors are sufficient to capture variation in total abundance, richness, and abundance distribution of taxa across microbiomes of different host types. However, each process, on its own, is unable to capture empirical realism. We used this theory to investigate known differences between two marine sponge host types, high and low microbial abundance (HMA and LMA), proposing ecological mechanisms that give rise to the group‐level separation observed across types. We have suggested that this baseline theory can be broadly applicable across diverse host‐microbiome systems. Relatively small modifications to quantitative values of the parameter controlling microbial dispersal were sufficient to investigate differences observed across human microbiomes. In this way we were able to generate hypotheses that help to explain differences observed between human palm and digestive system microbiomes. Our findings suggest the existence of general underlying ecological principles being key to the assembly of microbiomes across taxonomically diverse host organisms.

Local environmental conditions and the dispersal capacity of individuals and species are recognised as fundamental mechanisms driving biodiversity across ecological and spatial scales (Leibold et al. 2017, 2004). Our theory supports the accumulating evidence that these processes are fundamental for the assembly of microbial communities (Miller et al. 2018). In marine sponges, we showed that the trade‐off between reduced microbial immigration (via e.g., lower pumping rates) and enhanced control of the internal host environment enable the HMA strategy. Moreover, our results suggest that these same mechanisms are likely behind the maintenance of the human faecal microbiome and the differences observed with the more exposed palm microbiomes. Among the mechanisms studied, in the particular case of the sponge microbiome, the differences in resource allocation (i.e., how much of acquired resources are used by microbes) were the highest. As a consequence, this mechanism had the strongest effect on the emergence of distinct microbial communities across the HMA and LMA types, whereas host selection primarily refined these outcomes by improving quantitative agreement with empirical data (see Figure S2). The large difference in resource allocation across microbiome types (two orders of magnitude) highlights the importance of quantifying the relative strength of different mechanisms. This point is further supported by our results on human microbiomes, since changing only the order of magnitude of the microbial influx (same as the pumping rate for sponges) considerably affects the observable differences between community types. Future modelling efforts could explicitly incorporate the same ecological mechanisms across other host‐associated systems to evaluate how their relative quantitative contributions shape observed biodiversity patterns. Systematically varying their strengths and comparing simulations with observed patterns would help determine whether distinct communities can generally be explained by shifts along shared ecological axes. Complementary experimental work could quantify these contributions through controlled manipulations of dispersal rates, resource availability, or host filtering, combined with comparative assessment across systems, thereby guiding and helping in parameterising theoretical frameworks (as we used experimental work done on sponges to parameterise the mechanistic differences in our model). The key determinant is the magnitude of the gap between systems along a given mechanistic axis, not the absolute parameter values.

This suggests that resource allocation is critical to the emergence of differences between functionally important vs. more open microbiomes, as is the case in the HMA‐LMA dichotomy in marine sponges and the digestive system versus palm microbiomes in humans. Nonetheless, an interesting emergent property of the HMA sponges and the human digestive system, observed in both empirical data and simulations, is the lower variability in abundance distributions across replicate samples (Figures 3F and 4D), consistent with strong control within the host's internal environment. Model simulations also predict a faster microbiome assembly (i.e., shorter transient dynamics) in HMA compared to LMA sponges (Figure 1C), suggesting possible testable differences in priority effects and ontogenetic microbiome development. Faster assembly through rapid microbial enrichment may reduce colonisation by taxa arriving in later stages (Fukami 2015), reinforcing internal environmental control and contributing to more predictable microbiome composition. This effect is likely intensified by the higher prevalence of vertical symbiont transmission in HMA sponges (de Oliveira et al. 2020; Ribes et al. 2015), further distinguishing their assembly process from that of LMA sponges.

Host‐mediated selection of microbes is a complex process that may involve the host's immune system, biochemical interactions, defence molecules, or the creation of specialised niches that enrich or suppress specific microbial taxa (McLoughlin et al. 2016; Pita et al. 2018; Woodhams et al. 2020). We have modelled host‐mediated selection through a simplified enrichment function based on microbial resource production, an abstraction that captures the outcome of host selection without explicitly modelling its biological details. Despite this simplification, our results are robust to variation in the selection function, as shown in our sensitivity analysis. Qualitative diversity patterns are preserved across different functions, with exponents around two providing the best fit to empirical data (Figure S3), suggesting that enrichment of microbes involves some non‐linear selection process. This hypothesis could be tested experimentally by decoupling microbial functional contribution from reproductive success and quantifying their respective effects. Future modelling efforts may explore more detailed implementations of host selection to evaluate the impact of different underlying mechanisms.

In our model, microbial dispersal was purely horizontal: hosts acquired microbes from the environment and began assembly without an initial microbiome. However, vertical transmission, that is, the microbial transfer from parent to offspring, is a well‐documented process (Abdelfattah et al. 2023; Engelberts et al. 2022). It often involves the preferential transfer of specific microbial taxa, resulting in differential transmission (Bright and Bulgheresi 2010; Lurgi et al. 2019; Syukur et al. 2024). This mechanism may be essential for accurately capturing microbiome composition and functional inheritance and might be particularly relevant for the evolution of microbes and hosts (Lurgi et al. 2019; Pankey et al. 2022). Nonetheless, our results suggest that vertical transmission is not necessary to reproduce key patterns of richness and diversity across the communities we considered.

Despite the potential generality of our theory in explaining differences between microbiome types, it remains limited by its reliance on a priori specification of relevant ecological mechanisms. Our model does not account for how mechanistic distinctions emerge naturally. Divergence between microbiomes has likely arisen through evolutionary time (Roughgarden 2023; Sieber et al. 2021), thus opening an important avenue for future research: the evolutionary emergence of assembly mechanisms. For marine sponges, it has been hypothesised that HMA sponge lineages evolved multiple times independently from LMA sponge ancestors across diverse clades, representing a more recent strategy (Pankey et al. 2022). Future research could explore scenarios in which hosts and microbes present a phylosymbiotic signal, leading to the spontaneous emergence of distinct microbiome types. This would involve simulating the gradual divergence of host traits to identify conditions under which HMA‐like microbiomes arise as a discrete class rather than along a gradient of coexistence. Such a study could resolve the observed group‐level separation in microbiome diversity in more mechanistic detail, incorporating host phylogenetic branching and potentially linking to genetic distances among species (Griffiths et al. 2019). Moreover, ontogenetic host development is also a complex biological process that mediates the mechanisms of microbiome assembly (Metcalf et al. 2019), such as changes in resource requirements. Rather than explicitly modelling host developmental stages, our model treats these processes as generic drivers that can, in principle, vary through time. From this perspective, host development can be understood as introducing structured temporal variation in dispersal regimes, resource availability, and selective pressures, which would naturally extend the current framework. In the same way, distinct implementations of the same mechanisms (e.g., community coalescence as another form of dispersal) are also natural extensions to our framework.

Our study demonstrates that mathematical models of community assembly reveal drivers of complex microbiome emergence by incorporating differences in host type‐level ecological mechanisms. Specifically, we show that variation in community types, such as observed in HMA and LMA sponges and in palm versus gut human microbiomes, can be explained by a few mechanisms. These findings advance ecological theory, providing a mechanistic link between individual‐level processes and community‐level patterns in microbial communities, connecting host traits to microbial diversity. Our work underscores the central roles of microbial dispersal, host selection, and microbe–resource interactions. Furthermore, our results clarify the relative importance of processes maintaining complex microbiomes, supporting theoretical approaches to manipulate microbiomes, which can have practical applications to preserve host health and ecological communities (Ge et al. 2021; Peixoto et al. 2022).

## 4 Methods

### 4.1 Simulations

Microbial types are defined by the set of resources they consume and produce, that is, the matrices $\beta_{\mathrm{il}}$ and $\alpha_{\mathrm{il}}$. Focusing on host‐mediated mechanisms, we standardised microbial traits across types, having constant intrinsic reproduction rates ($w_{0}$), death rates ($d_{0}$), and intraspecific competition coefficient (γ).

Each microbial type consumes $n_{c}=100$ resources and produces $n_{p}=100$ metabolic products. Resource production is uniformly distributed across selected products, with no preference for resource types. Thus, nonzero entries of the production matrix α are $1/n_{p}$.

To produce Figures 2 and 3, we simulated 24 sponge species divided into 12 HMA and 12 LMA species, each with 6 individuals (in the model, hosts are dynamically independent from each other, so host species richness influences only the representation of host variation). The number of resources was R=5000 and of microbes S=3000. They were equally separated into the three groups.

4.2

Parameter values used in the main simulations were as follows: $w_{0}=5$, ϵ=0.7, $d_{0}=0.5$, γ=1, $\delta_{0}=0.1$. $\beta_{\mathrm{il}}∼U\left[0,10\right]$ for consumed resources and zero otherwise, $\rho_{l}∼30*\text{Lognormal}\left(0,1\right)$, and $\chi_{i}=0.0001U_{h}\left[0,1\right]$. $c_{\mathrm{lh}}=U_{l}\left[0,1\right]\left(1+N_{h}\left(0,0.1\right)\right)$ for primary resources and zero for others (the same for individuals of the same species). Pumping rates were $\mu_{h}=0.0002\left(1+0.5U_{h}\left[-0.5,0.5\right]\right)$ for HMA samples and 2.5 times larger for LMA samples. Resource allocation was $\eta_{h}=0.5\left(1+0.5U_{h}\left[-0.5,0.5\right]\right)$ for HMA samples and $\eta_{h}=0.005\left(1+0.5U_{h}\left[-0.5,0.5\right]\right)$ for LMA samples. Selection capacity was $v_{h}=0.2$ for HMA samples and $v_{h}=0.01$ for LMA samples. Every sponge species valued the same $q_{1}=15$ essential resources and each species valued on average $q_{2}=15$ non‐essential resources (the chance of valuing non‐essential resources is $q_{2}/\xi$, where $\xi =\left(2R/3\right)-q_{1}$ represents non‐essential secondary/tertiary resources).

All hosts started with initial abundances $x_{\mathrm{ih}}^{0}=0$, and the same for resources. We integrated the model for 400 periods, each with 10 timesteps of size 0.01, and analysed the model's final state.

In Figure 4, we simulated 24 sample types (analogous to sponge species) divided into 12 palm and 12 faeces types. Each type had 6 individuals. The only difference from previous simulations was that microbial dispersal (same as pumping rates, $\mu_{h}$) of palms was 10 times that of faeces.

Simulations were performed in Python (v3.11.13; Van Rossum and Drake 1995) using NumPy (v2.0.1; Harris et al. 2020) and the function *integrate.odeint* from SciPy (v1.16.0; Virtanen et al. 2020).

### 4.3 Sponge Microbiome Data

We used the microbiome data of operational taxonomic units (OTU) from the Sponge Microbiome Project (Moitinho‐Silva, Nielsen, et al. 2017). Moitinho‐Silva, Steinert, et al. (2017) used this data and presented a table of sponge sample IDs and their classification as HMA or LMA sponges based on electron microscopy analyses (Gloeckner et al. 2014), which we used to select sponge species and their OTU composition. We also removed OTUs appearing in less than 10 counts over the entire table. The final table comprises 496 samples (312 HMA sponges and 184 LMA sponges) separated into 36 species (19 HMA sponges and 17 LMA sponges). We rarefied the OTU data with the minimum dataset depth of 23,376. The pool of OTUs in the final data were 56,161.

### 4.4 Human Microbiome Data

We used data publicly available from EBI Metagenomics: longitudinal human microbiome data collected over 396 timepoints (faeces and left/right palm; subject M3; Study MGYS00002184) from the EBI MGnify platform (project ERP021896) (Caporaso et al. 2011; Mitchell et al. 2018). From this project we obtained an OTU table on which OTUs were defined at a 97% similarity and generated using the bioinformatics pipeline described in (Caporaso et al. 2011) using Quantitative Insights Into Microbial Ecology (QIIME, Caporaso et al. 2010). We considered samples having at least 20,000 reads, applying rarefaction accordingly. We obtained 330 for faeces, and 265 and 20 for each palm.

### 4.5 Analysis

We sampled 24,000 counts using the model's final microbial abundances as weights and created OTU tables equivalent to the SMP data for direct comparison (we used 20,000 counts for the human microbiome analysis).

We created a Bray–Curtis distance matrix of log‐transformed relative microbial abundances, using R (v4.5.0; R Core Team 2021), and applied a nonmetric multidimensional scaling (NMDS) analysis, using *decostand*, *log1p* and *metaMDS* from R's *Vegan* (Dixon 2003). Comparing the spread in NMDS projection between groups, we calculated points' mean distances to the group's centroid (average position) for each group and analysed their ratio.

For additional evidence of group separation, we performed a PERMANOVA on Bray–Curtis dissimilarity matrices of HMA and LMA sponge samples, using *vegdist* and *adonis2* from R's *Vegan* (Dixon 2003).

As a measure of diversity, we calculated the evenness of OTU distributions of each sample (normalised Shannon entropy of the distribution of relative abundances). Therefore, $E=\frac{H^{\prime }}{\log S^{\prime }}$, with $H^{\prime }=-\sum_{i=1}^{S}p_{i}\log p_{i}$ for the relative abundances $p_{i}$. *S* = number of non‐zero counts of OTUs.

To test group separation of mean richness and evenness between LMA and HMA (and between palm and faeces), we performed a Welch's *t*‐test for two groups with unequal variances.

We ranked OTUs and plotted their ranked abundances, from maximum to minimum, showing relative abundances in the range $\left[10^{-4},1\right]$. Curves show average values with shaded regions representing standard deviations for each rank.

To compare variability in evenness between empirical and simulated datasets, we assessed differences in variance using Levene's test (Brown–Forsythe variant, centered on the median), which is robust to deviations from normality (Brown and Forsythe 1974). Tests were performed between groups (palm and faeces) across empirical and simulated distributions.

## Author Contributions

M.L., T.T., J.M.M., N.S.W. and G.A. conceived and designed the study. G.A. implemented the model and performed numerical simulations and analysis. M.L. and G.A. wrote the manuscript. M.L., T.T., J.M.M., N.S.W. and G.A. revised the manuscript.

## Supporting information


**Figure S1:** Empirical patterns of marine sponges in a subsample of the data. A random subsample of the marine sponge data presented in Figures 2 and 3 with a homogeneous sampling between groups: 7 HMA and 7 LMA species with 6 samples each. (A) Microbial richness distributions, (B) evenness distributions, (C) rank‐abundance plot and (D) NMDS ordination showing the group separation. See captions of Figures 2 and 3 for details.
**Figure S2:** Separate effects of mechanisms. Model parameters and plots are the same as presented in Figures 2 and 3. HMA sponges follow a trade‐off between lower pumping rates and better ‘cultivating’ the microbiome. Differences in all mechanisms together are required to produce a good representation of the data. (A) Here, the difference between HMA and LMA is only that LMAs have 2.5 higher pumping rates. This results in reversed patterns, with HMA displaying lower abundance, richness, and evenness. (B) Then, on top of the differences in pump rates, considering resource allocation alone (100 times higher for HMA), the patterns are qualitatively well‐represented, but the abundance ratio is lower and other differences are exaggerated. (C) Considering only selection on top of pumping rate (HMA selection capacity of 0.2 and LMA of 0.01), without differences in resource allocation, the patterns do not reflect observation of natural microbiomes. HMA richness becomes very low and total abundances are roughly on the same level. For this analysis, we simulated 6 LMA and 6 HMA species, each with 3 samples.
**Figure S3:** Sensitivity analysis on the selection function. Model parameters and plots are the same as presented in Figures 2 and 3. Each row shows results for a particular curve, defined by the selection exponent y in Equation (3) with value equal to 2 in the main model. Values are (A) y=0.5, (B) y=1 (linear), (C) y=1.5, (D) y=2.5 and (E) y=3. For this analysis, we simulated 6 LMA and 6 HMA species, each with 3 samples.
**Figure S4:** Sensitivity analysis on the resource values. Model parameters and plots are the same as presented in Figures 2 and 3. Each row shows results for a particular configuration of fixed and random resources valued by hosts (as explained in Box 2). The main model uses ($q_{1}=15,q_{2}=15$) and we tested different proportions and values: (A) ($q_{1}=15,q_{2}=0$), (B) ($q_{1}=15,q_{2}=30$), (C) ($q_{1}=5,q_{2}=15$), (D) ($q_{1}=30,q_{2}=15$) and (E) ($q_{1}=30,q_{2}=30$). For this analysis, we simulated 6 LMA and 6 HMA species, each with 3 samples.
**Figure S5:** Sensitivity analysis on the total number of microbial types (S). Model parameters and plots are the same as presented in Figures 2 and 3. Each row shows results for different values of S. The main model uses S=3000, and we tested different values: (A) S=1000, (B) S=1500, (C) S=2000 and (D) S=2500. For this analysis, we simulated 6 LMA and 6 HMA species, each with 3 samples.
**Figure S6:** Detailed distributions for human microbiome data. The same analysis summarised in Figure 3 but shown for each microbiome separately. Groups belong to a particular microbiome of a single individual, with different samples taken in different moments (timeseries). (A, B) Show, respectively distributions of richness and evenness.
**Figure S7:** Sensitivity analysis on the dispersal differences in human simulations. Model parameters and plots are the same as presented in Figure 4. Each row shows results for different values of the gap in dispersal between the two types. The main model makes dispersal for the palm group 10 times higher than the faeces group (baseline), and we tested different values: (A) 5 times higher, (B) 7.5 times higher, (C) 12.5 times higher and (D) 15 times higher. For this analysis, we simulated 6 palm and 6 faeces variants, each with 3 samples.

## Data Availability

All datasets and computer code developed and used in this study are available in the accompanying Zenodo repository https://doi.org/10.5281/zenodo.17107189. Computer source code for the implementation of the model is also available on GitHub: https://github.com/computational‐ecology‐lab/microbiome‐assembly‐principles. Additionally, computer code and a tutorial notebook to reproduce a minimal example of our model simulations is available at: https://github.com/computational‐ecology‐lab/microbiome‐assembly‐principles/tree/main/Simple_code_example and on the Zenodo repository.
