# Supplementary material for: Towards Key Principles of Host‐Associated Microbiome Assembly
Source: Ecol Lett. 2026 Jun 24;29(6):e70433. doi: 10.1111/ele.70433 (PMC13291775; doi:10.1111/ele.70433)
Supplement: Supplementary file 1 — Figure S1: Empirical patterns of marine sponges in a subsample of the data. A random subsample of the marine sponge data presented in Figures 2 and 3 with a homogeneous sampling between groups: 7 HMA and 7 LMA species with 6 samples each. (A) Microbial richness distributions, (B) evenness distributions, (C) rank‐abundance plot and (D) NMDS ordination showing the group separation. See captions of Figures 2 and 3 for details. Figure S2: Separate effects of mechanisms. Model parameters and plots are the same as presented in Figures 2 and 3. HMA sponges follow a trade‐off between lower pumping rates and better ‘cultivating’ the microbiome. Differences in all mechanisms together are required to produce a good representation of the data. (A) Here, the difference between HMA and LMA is only that LMAs have 2.5 higher pumping rates. This results in reversed patterns, with HMA displaying lower abundance, richness, and evenness. (B) Then, on top of the differences in pump rates, considering resource allocation alone (100 times higher for HMA), the patterns are qualitatively well‐represented, but the abundance ratio is lower and other differences are exaggerated. (C) Considering only selection on top of pumping rate (HMA selection capacity of 0.2 and LMA of 0.01), without differences in resource allocation, the patterns do not reflect observation of natural microbiomes. HMA richness becomes very low and total abundances are roughly on the same level. For this analysis, we simulated 6 LMA and 6 HMA species, each with 3 samples. Figure S3: Sensitivity analysis on the selection function. Model parameters and plots are the same as presented in Figures 2 and 3. Each row shows results for a particular curve, defined by the selection exponent y in Equation (3) with value equal to 2 in the main model. Values are (A) y=0.5, (B) y=1 (linear), (C) y=1.5, (D) y=2.5 and (E) y=3. For this analysis, we simulated 6 LMA and 6 HMA species, each with 3 samples. Figure S4: Sensitivity analysis on [file ELE-29-0-s001.docx]

**Supplementary Material for the paper:**

**“Towards key principles of host-associated microbiome assembly”**

Gui Araujo^1^, Torsten Thomas^2^, José M Montoya^3^, Nicole S Webster^4,5^ & Miguel Lurgi^1,*^

^1^Department of Biosciences, Swansea University. Swansea, SA2 8PP. UK.

^2^Centre for Marine Science and Innovation, School of Biological, Earth & Environmental Sciences, University of New South Wales. Sydney, 2052, Australia.

^3^Theoretical and Experimental Ecology Station, CNRS. 2 route du CNRS, 09200 Moulis, France.

^4^Institute for Marine and Antarctic Studies, University of Tasmania. Hobart, 7001, Australia.

^5^Australian Centre for Ecogenomics, University of Queensland, Brisbane, 4072, Australia

* corresponding author: [miguel.lurgi@swansea.ac.uk](mailto:miguel.lurgi@swansea.ac.uk)


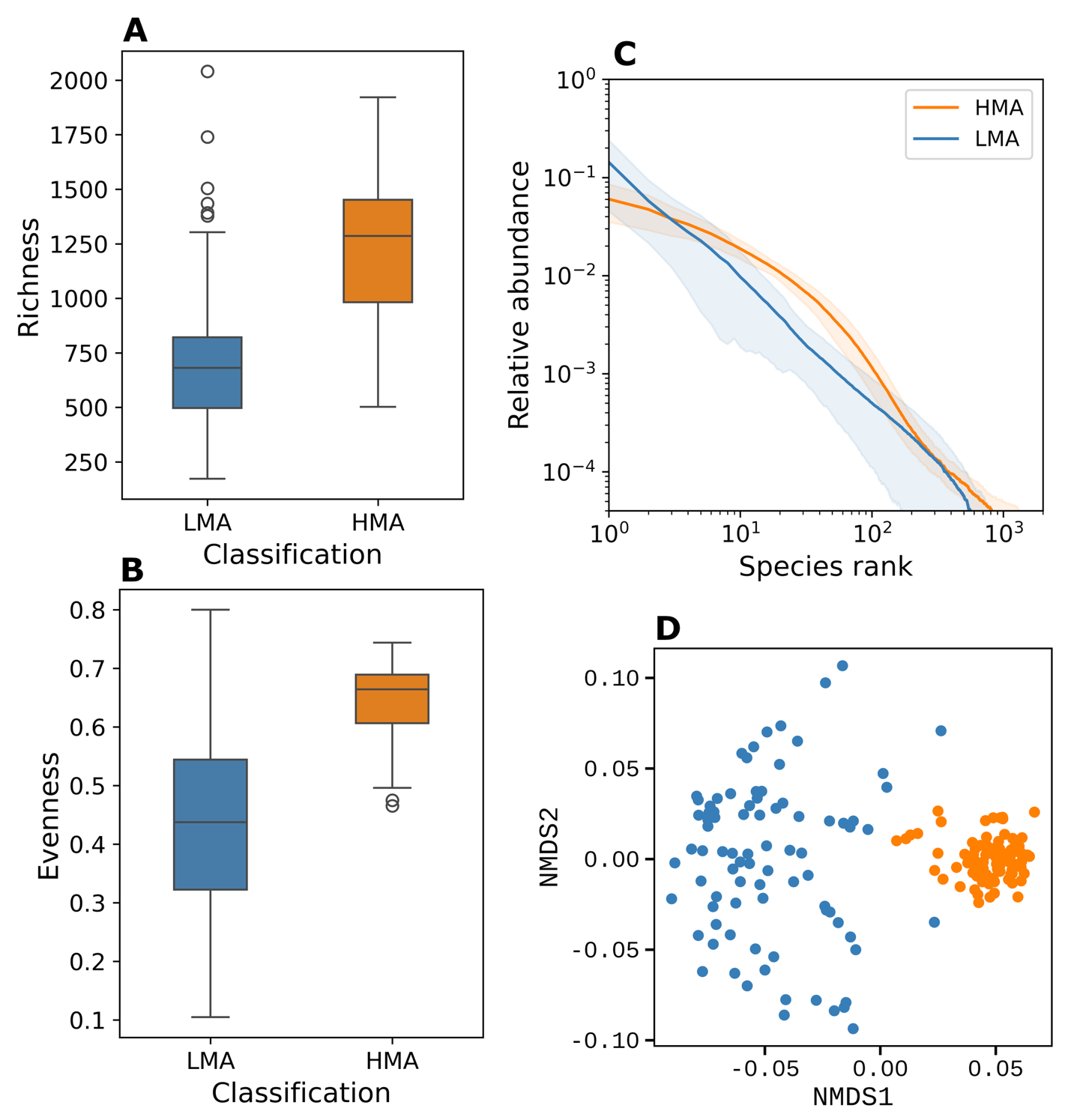


Supplementary Figure 1. **Empirical patterns of marine sponges** **in a subsample of the data**. A random subsample of the marine sponge data presented in Figs 2 and 3 with a homogeneous sampling between groups: 7 HMA and 7 LMA species with 6 samples each. (A) Microbial richness distributions, (B) evenness distributions, (C) rank-abundance plot, and (D) NMDS ordination showing the group separation. See captions of Figs 2 and 3 for details.


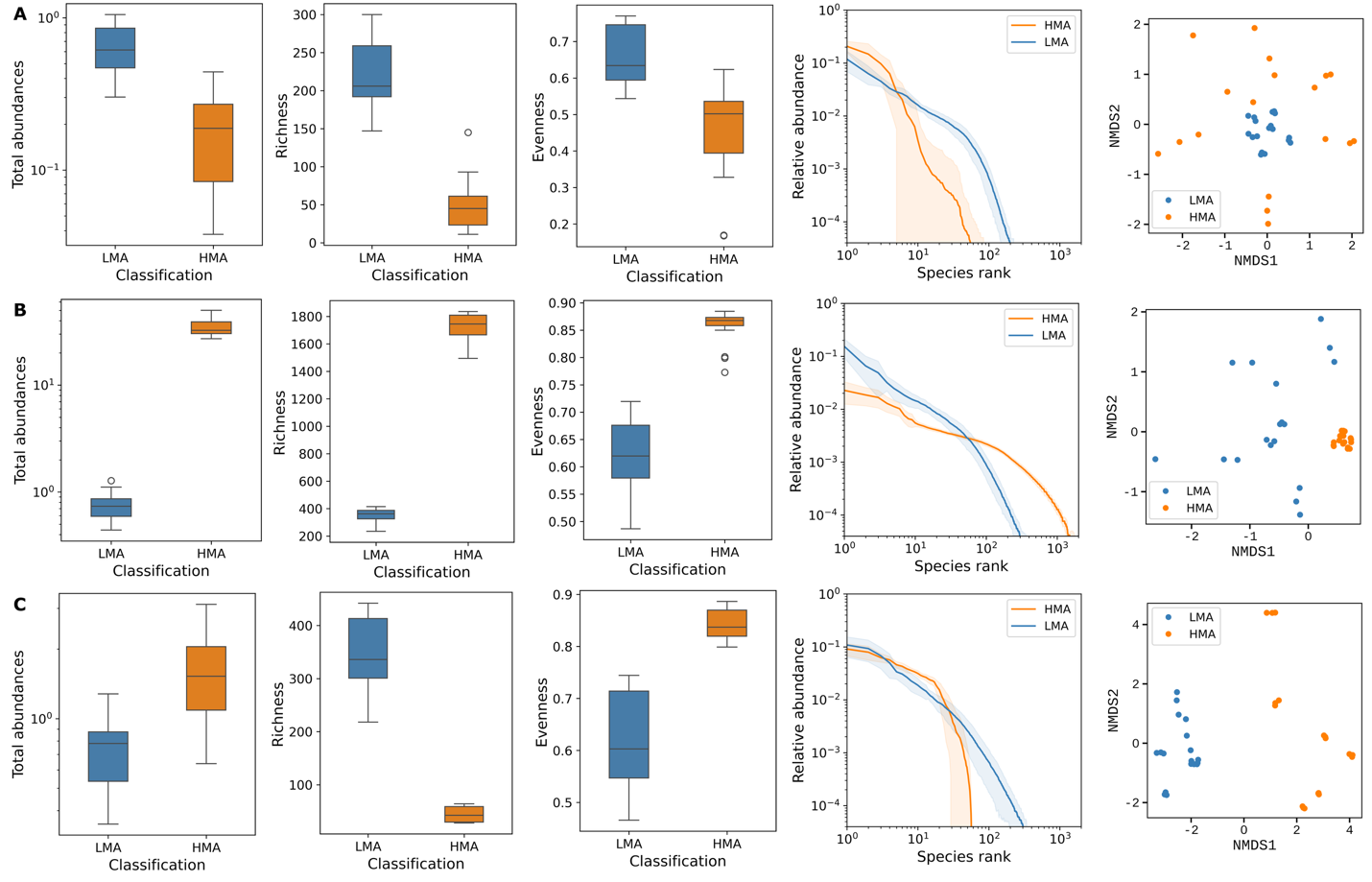


Supplementary Figure 2. **Separate effects of mechanisms**. Model parameters and plots are the same as presented in Figs. 2 and 3. HMA sponges follow a trade-off between lower pumping rates and better “cultivating” the microbiome. Differences in all mechanisms together are required to produce a good representation of the data. (A) Here, the difference between HMA and LMA is only that LMAs have 2.5 higher pumping rates. This results in reversed patterns, with HMA displaying lower abundance, richness, and evenness. (B) Then, on top of the differences in pump rates, considering resource allocation alone (100 times higher for HMA), the patterns are qualitatively well-represented, but the abundance ratio is lower and other differences are exaggerated. (C) Considering only selection on top of pumping rate (HMA selection capacity of 0.2 and LMA of 0.01), without differences in resource allocation, the patterns do not reflect observation of natural microbiomes. HMA richness becomes very low and total abundances are roughly on the same level. For this analysis, we simulated 6 LMA and 6 HMA species, each with 3 samples.


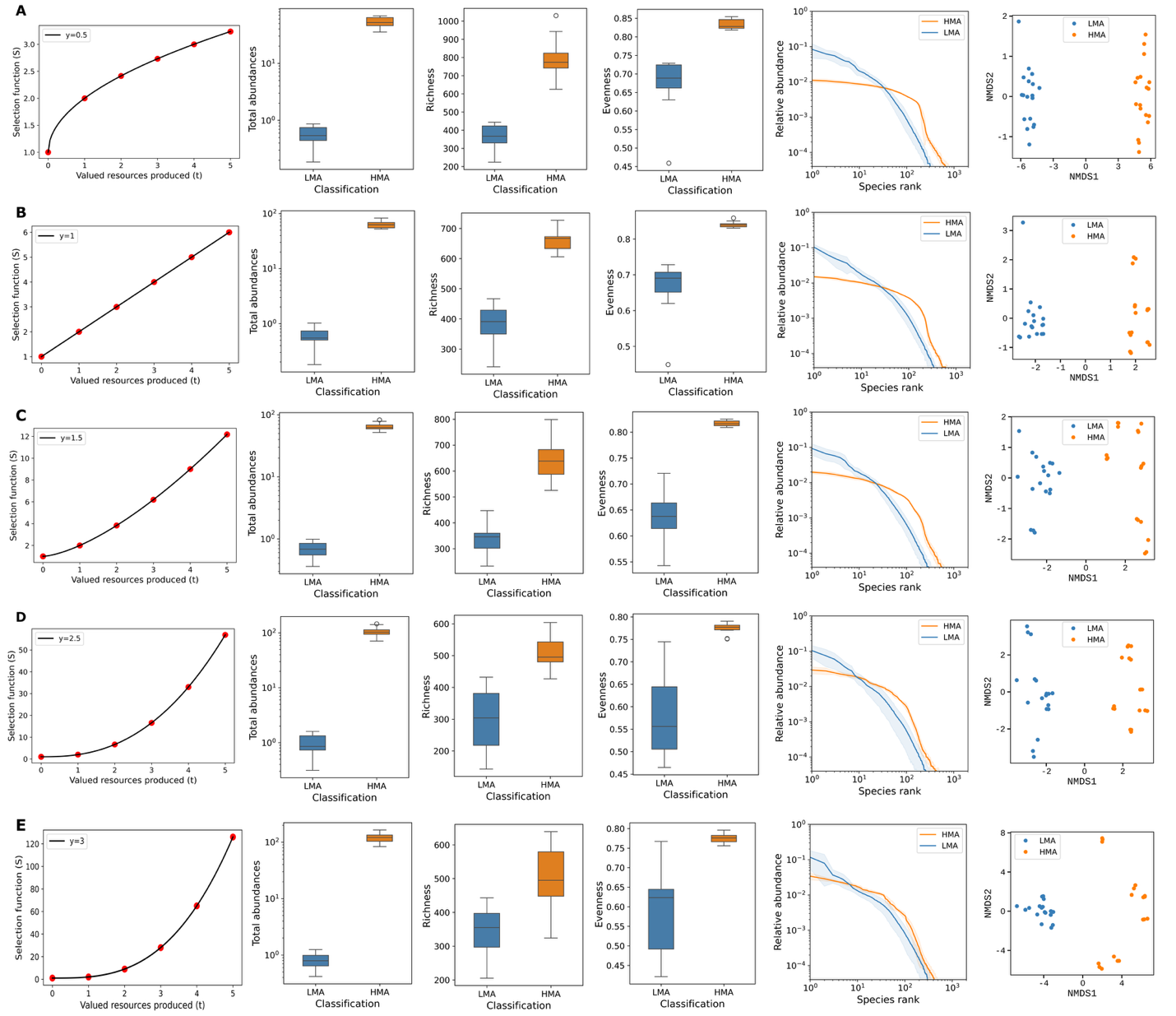


Supplementary Figure 3. **Sensitivity analysis on the selection function**. Model parameters and plots are the same as presented in Figs. 2 and 3. Each row shows results for a particular curve, defined by the selection exponent $y$ in Eq 3 with value equal to 2 in the main model. Values are (A) $y=0.5$, (B) $y=1$ (linear), (C) $y=1.5$, (D) $y=2.5$, and (E) $y=3$. For this analysis, we simulated 6 LMA and 6 HMA species, each with 3 samples.


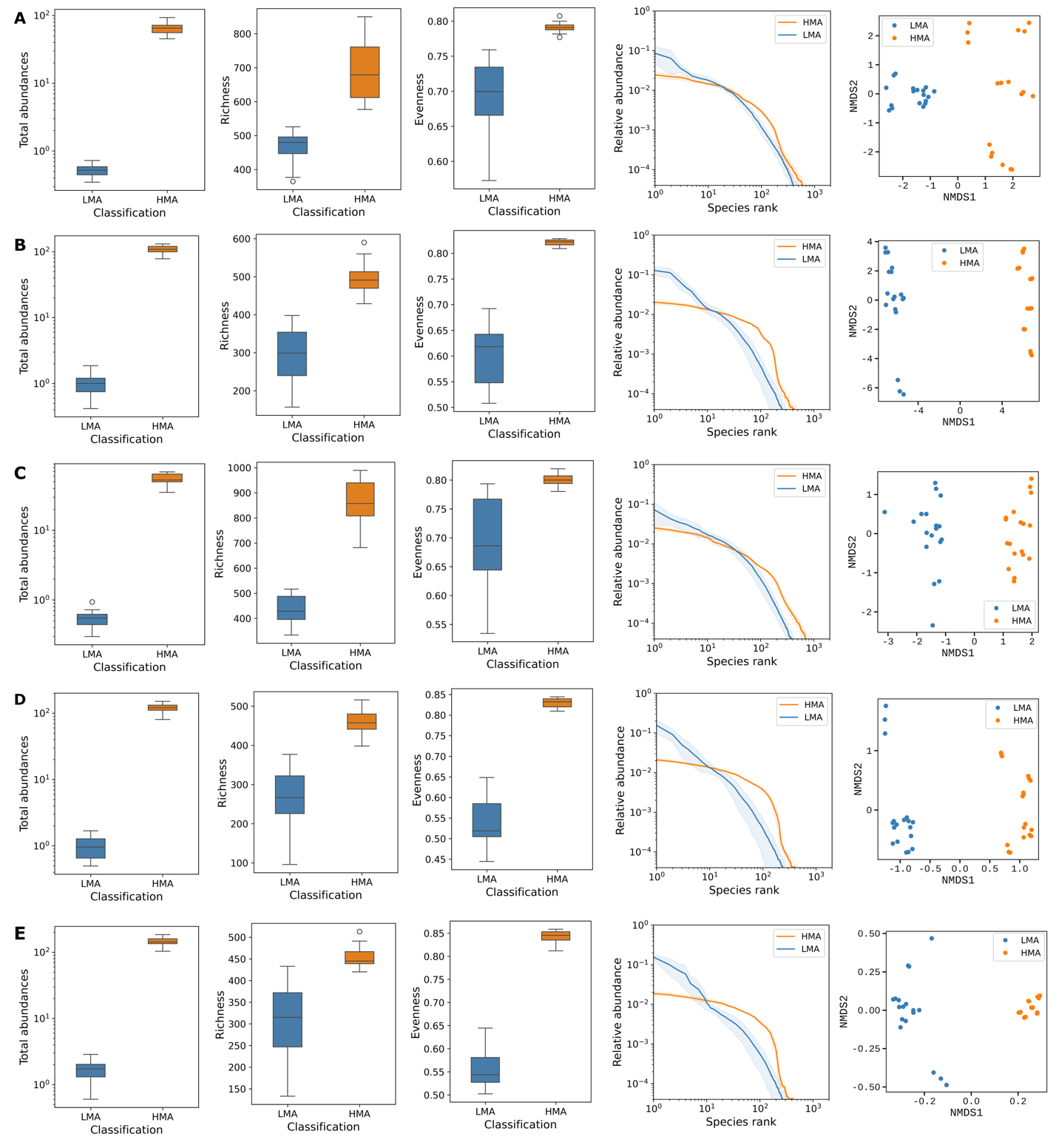


Supplementary Figure 4. **Sensitivity analysis on the resource values**. Model parameters and plots are the same as presented in Figs. 2 and 3. Each row shows results for a particular configuration of fixed and random resources valued by hosts (as explained in Box 2). The main model uses ($q_{1}=15,q_{2}=15$) and we tested different proportions and values: (A) ($q_{1}=15,q_{2}=0$), (B) ($q_{1}=15,q_{2}=30$), (C) ($q_{1}=5,q_{2}=15$), (D) ($q_{1}=30,q_{2}=15$), and (E) ($q_{1}=30,q_{2}=30$). For this analysis, we simulated 6 LMA and 6 HMA species, each with 3 samples.


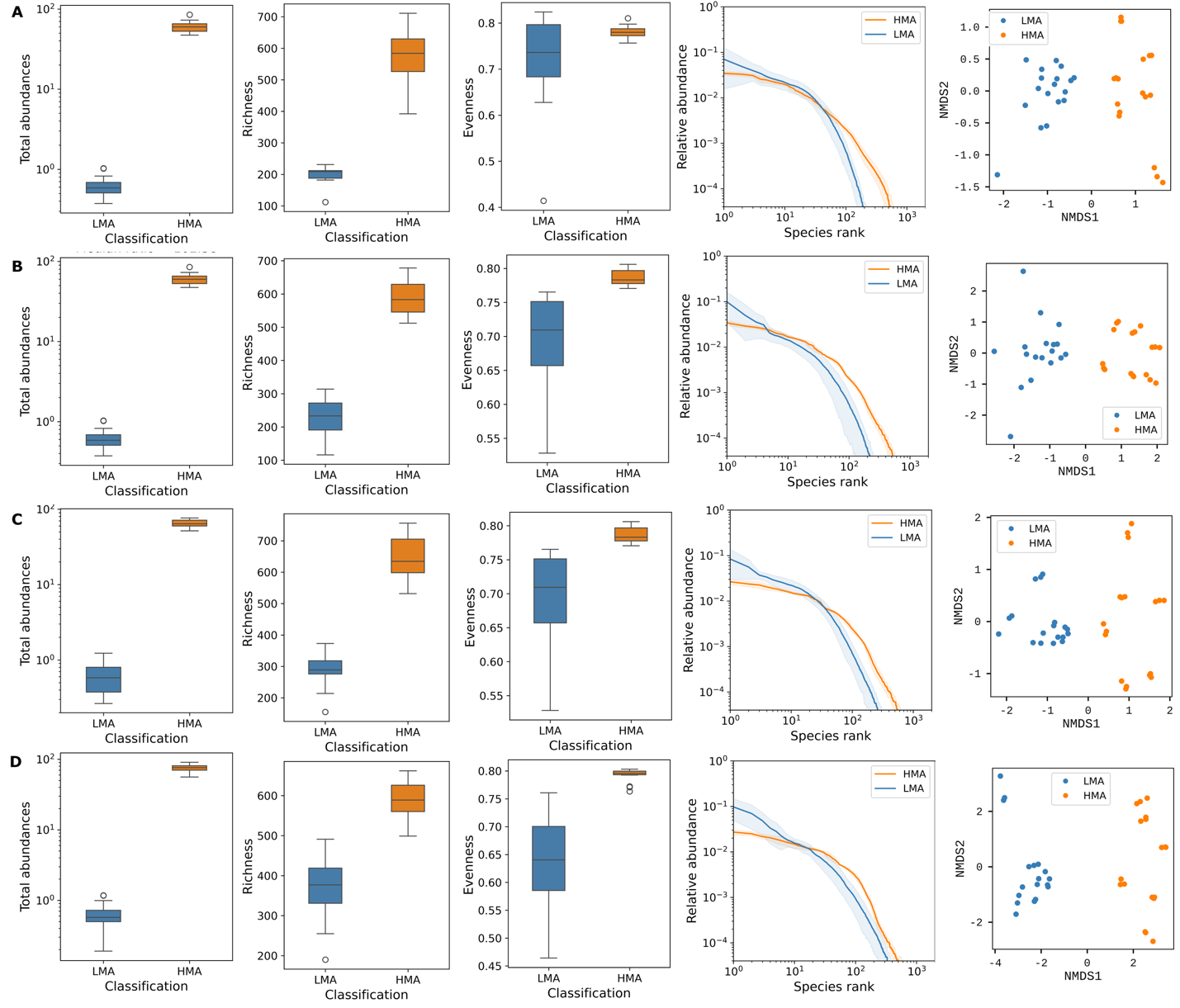
Supplementary Figure 5. **Sensitivity analysis on the total number of microbial types (**$\boldsymbol{S}$**).** Model parameters and plots are the same as presented in Figs. 2 and 3. Each row shows results for different values of $S$. The main model uses $S=3,000$, and we tested different values: (A) $S=1,000$, (B) $S=1,500$, (C) $S=2,000$, and (D) $S=2,500$. For this analysis, we simulated 6 LMA and 6 HMA species, each with 3 samples.

**
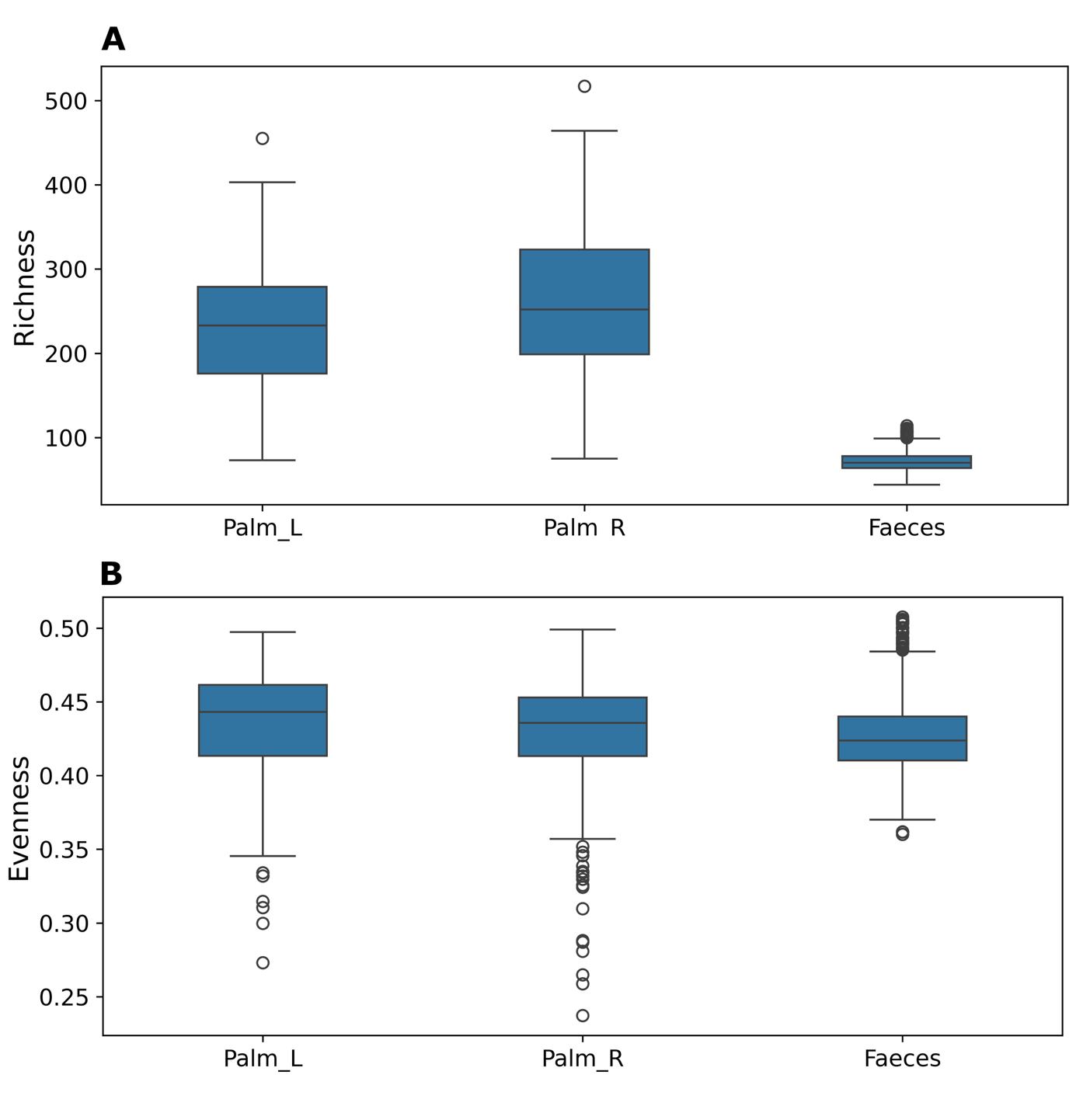
**Supplementary Figure 6. **Detailed distributions for human microbiome data**. The same analysis summarised in Fig. 3 but shown for each microbiome separately. Groups belong to a particular microbiome of a single individual, with different samples taken in different moments (timeseries). (A) and (B) show respectively distributions of richness and evenness.


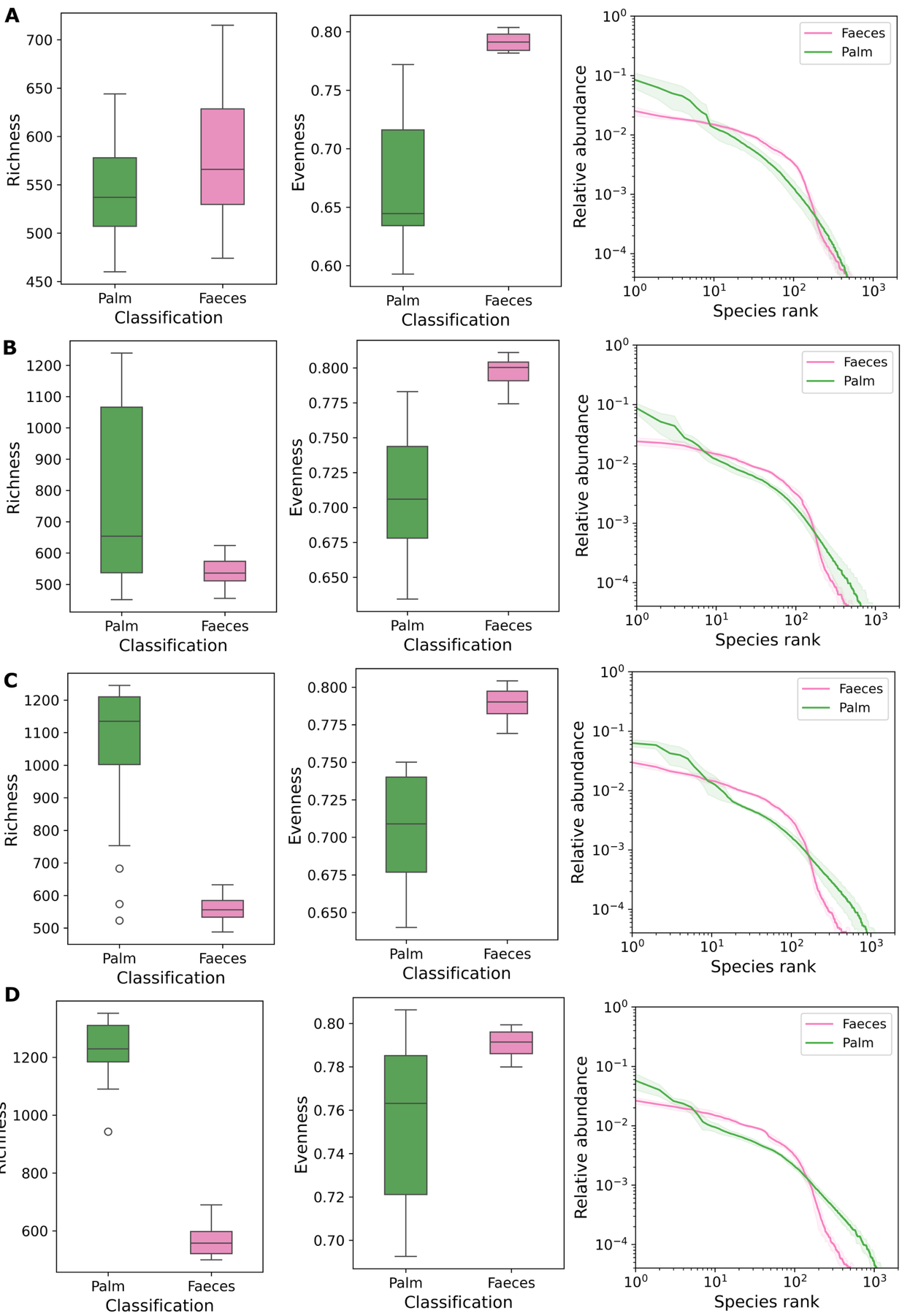
Supplementary Figure 7. **Sensitivity analysis on the dispersal differences in human simulations**. Model parameters and plots are the same as presented in Fig. 4. Each row shows results for different values of the gap in dispersal between the two types. The main model makes dispersal for the palm group 10 times higher than the faeces group (baseline), and we tested different values: (A) 5 times higher, (B) 7.5 times higher, (C) 12.5 times higher, and (D) 15 times higher. For this analysis, we simulated 6 palm and 6 faeces variants, each with 3 samples.
